# Incomplete Kawasaki Disease Presenting With Coronary Artery Aneurysms in a 2.5-Month-Old Infant: A Case Report

**DOI:** 10.7759/cureus.103981

**Published:** 2026-02-20

**Authors:** Zain H Haddad, Yousef Basma

**Affiliations:** 1 General Practice, University Hospital Sharjah, Sharjah, ARE; 2 Pediatrics, University Hospital Sharjah, Sharjah, ARE

**Keywords:** aspirin, coronary artery ectasia, echocardiography, incomplete kawasaki disease, infants, intravenous immunoglobulin, kawasaki disease

## Abstract

Kawasaki disease (KD) is an acute systemic vasculitis of childhood and remains a leading cause of acquired heart disease in children. Diagnosis in infants younger than six months is particularly challenging because presentations are often incomplete and may mimic common infectious conditions, resulting in delayed recognition and an increased risk of coronary artery complications. We report a case of a 2.5-month-old female infant who presented with persistent fever and nonspecific upper respiratory symptoms and was initially treated for a presumed infectious etiology. Despite the absence of classic mucocutaneous features, progressive systemic inflammation with anemia and thrombocytosis, a negative infectious evaluation, and echocardiography demonstrating coronary artery abnormalities within the aneurysm range by Z-score thresholds (maximum Z-scores: left main coronary artery (LMCA) +5.5, left anterior descending artery (LAD) +6.9, right coronary artery (RCA) +6.2, and left circumflex artery (LCx) +3.7) supported the diagnosis of incomplete Kawasaki disease (iKD).

The patient received intravenous immunoglobulin (2 g/kg) and aspirin therapy, with rapid defervescence and improvement in inflammatory markers. Follow-up echocardiography approximately six weeks later demonstrated marked interval improvement in coronary artery dimensions, with a reduction in Z-scores. This report highlights the need for a high index of suspicion for iKD in very young infants with persistent fever and systemic inflammation and underscores the importance of early echocardiography to enable timely treatment and reduce potentially preventable coronary complications.

## Introduction

Kawasaki disease (KD) is an acute, self-limited systemic vasculitis of unknown etiology that predominantly affects medium-sized arteries, with a particular predilection for the coronary arteries [1,2]. It remains a leading cause of acquired heart disease in children in developed countries [1,2]. Timely recognition and treatment are essential, as coronary artery abnormalities, including dilatation and aneurysm formation, develop in approximately 20-25% of untreated patients and can lead to long-term cardiovascular morbidity [1,2].

The diagnosis of KD is based primarily on clinical findings. Classic KD is typically defined by fever for ≥5 days accompanied by at least four of five principal features (bilateral non-exudative conjunctival injection, oral mucosal changes, polymorphous rash, extremity changes, and cervical lymphadenopathy), whereas incomplete Kawasaki disease (iKD) presents with fewer than four features and therefore frequently necessitates supportive laboratory evidence of systemic inflammation and echocardiographic findings after exclusion of alternative causes of fever [1,2]. Early presentations can be nonspecific and may resemble common infectious conditions, leading to initial antibiotic therapy and delayed consideration of inflammatory etiologies [3,4]. In the current era, multisystem inflammatory syndrome in children (MIS-C) is also an important inflammatory mimic that may manifest with persistent fever, elevated inflammatory markers, and cardiac involvement, requiring careful clinical and laboratory correlation when clinical features overlap [5].

Early transthoracic echocardiography is crucial in the evaluation of suspected KD. Contemporary guidance emphasizes the importance of using body surface area-adjusted coronary artery Z-scores for diagnosing and classifying coronary artery abnormalities and for ensuring consistent follow-up across patient size and age [6]. Infants younger than six months often present with iKD, which makes early diagnosis challenging and often delayed [1,2,7]. Echocardiography is particularly valuable in iKD because coronary abnormalities may exist even when classic mucocutaneous signs are absent [8].

Regional epidemiologic data from the United Arab Emirates highlight the clinical relevance of incomplete KD and its coronary outcomes, emphasizing the need for increased vigilance in children with persistent fever and systemic inflammation even in the absence of classic mucocutaneous findings [9]. Infants, particularly those younger than six months, are at higher risk of developing coronary artery abnormalities than older children, and persistent unexplained fever may be the predominant presenting feature; delayed diagnosis in this age group can contribute to worse coronary outcomes [10,11]. This report describes a case of incomplete KD in a 2.5-month-old infant with prolonged fever and minimal clinical features who was initially treated for a suspected infection and subsequently found to have coronary artery abnormalities fulfilling aneurysm-range Z-score criteria.

## Case presentation

A 2.5-month-old female infant, born at term (39 weeks’ gestation) with an unremarkable perinatal history and normal Apgar scores, presented with fever and mild upper respiratory symptoms. She had been previously well, with no significant past medical history. The initial presentation occurred on October 14, 2025 (day one of illness; fever onset), when she developed fever with a maximum recorded temperature of 38 °C, accompanied by mild cough and nasal congestion. There was no history of poor feeding, vomiting, diarrhea, rash, conjunctivitis, oral mucosal changes, extremity swelling, or lymphadenopathy. On examination, the infant appeared clinically well, with stable vital signs and no focal abnormalities. Cardiovascular examination revealed normal heart sounds without an audible murmur. Initial laboratory investigations demonstrated anemia and elevated inflammatory markers (Table 1).

**Table 1:** Serial laboratory investigations during the course of illness Serial laboratory trends during the course of illness. Reference ranges are shown in the column headers WBC: white blood cell count; CRP: C-reactive protein; ESR: erythrocyte sedimentation rate; IVIG: intravenous immunoglobulin. “—” indicates a value that was not measured at that time point

Date (2025)	Clinical stage	Hemoglobin (g/dL) (Ref 11.1–14.1)	WBC (×10⁹/L) (Ref 6.0–18.0)	Platelets (×10⁹/L) (Ref 200–550)	CRP (mg/L) (Ref 0–8)	ESR (mm/hr) (Ref 0–20)
14/10	Initial presentation	8.9	10.72	589	18.9	—
21/10	Readmission/diagnostic escalation	7.8	16.94	1076	80.3	—
23/10	Post-IVIG (acute phase)	7.1	9.11	1126	66.7	45
25/10	Early improvement	7.9	10.27	1243	18.4	34
20/11	Outpatient follow-up	11.1	9.47	503	—	—

Respiratory viral panel and influenza testing were negative. Urinalysis showed leukocyturia with urine WBC 11-20/hpf (reference: 0-5/hpf), and leukocyte esterase 1+ (reference: negative), and urine culture yielded mixed growth. In view of a suspected bacterial infection, possibly urinary tract infection, the patient was treated with intravenous ceftriaxone (300 mg IV once daily for three days) and supportive care. She remained clinically stable and was discharged home on oral cephalosporins (30 mg orally once daily for three days) on October 19, 2025. On October 21, 2025 (day eight of illness, from fever onset), the patient re-presented to the outpatient clinic with persistent intermittent fever. Repeat laboratory investigations showed worsening inflammation, progressive leukocytosis, marked thrombocytosis, and worsening anemia (Table 1). Procalcitonin remained within normal limits (0.14 ng/mL, reference less than 0.5 ng/mL).

Given concern for ongoing sepsis or possible central nervous system infection, the patient was re-admitted on October 21, 2025, for further evaluation. On re-admission, the infant appeared irritable but was feeding adequately. Physical examination revealed mild pallor without cyanosis or jaundice. There was no rash, conjunctival injection, oral mucosal changes, extremity edema, or lymphadenopathy. Respiratory examination was unremarkable, and there were no signs of meningeal irritation. Lumbar puncture was performed as part of the evaluation of a febrile young infant to exclude central nervous system infection, such as meningitis, in the setting of persistent fever and ongoing systemic inflammation. Cerebrospinal fluid (CSF) analysis showed a mildly elevated white blood cell count (13/µL) with mononuclear predominance, mildly elevated protein (640 mg/L), and normal glucose, with no organisms seen on Gram stain. These CSF findings, together with negative microbiologic testing, reduced the likelihood of bacterial meningitis and supported continued evaluation for alternative inflammatory etiologies (Table 2).

**Table 2:** CSF analysis CSF: cerebrospinal fluid; PCR: polymerase chain reaction

Parameter	Result	Reference range
Appearance	Clear, colorless	Clear
White blood cells	13 cells/µL	0–7 cells/µL
Red blood cells	5 cells/µL	0–50 cells/µL
Neutrophils	5%	0–8%
Lymphocytes	38%	2–38%
Monocytes	56%	50–94%
Eosinophils	1%	Not established
Protein	640 mg/L	150–450 mg/L
Glucose	3.5 mmol/L	3.3–4.4 mmol/L
Gram stain	No organisms seen	Negative
CSF culture	No growth at 72 hours	No growth
Meningitis PCR panel	Negative for all targets	Negative

Blood and urine cultures remained negative. CSF culture was likewise negative. Laboratory evaluation at this stage demonstrated progressive anemia, significant thrombocytosis, elevated inflammatory markers, and leukocytosis (Table 1). Additional investigations showed ferritin of 289.30 ng/mL (reference: 24-307 ng/mL), high-sensitivity troponin I at 7.79 ng/L (reference: 0.00-47.30 ng/L), lactate dehydrogenase 198 U/L (reference: 120-246 U/L), and NT-proBNP 413 pg/mL (adult reference range: 0-125 pg/mL). The laboratory reference range was derived from adult values, whereas NT-proBNP levels are physiologically elevated in early infancy; this result was therefore interpreted in that context. Urea was 3.80 mmol/L (reference: 3.20-8.20 mmol/L). Liver enzymes were within reference ranges (ALT: 19 U/L [reference: 14-59 U/L], AST: 29 U/L [reference: 8-34 U/L]).

**Figure 1:**
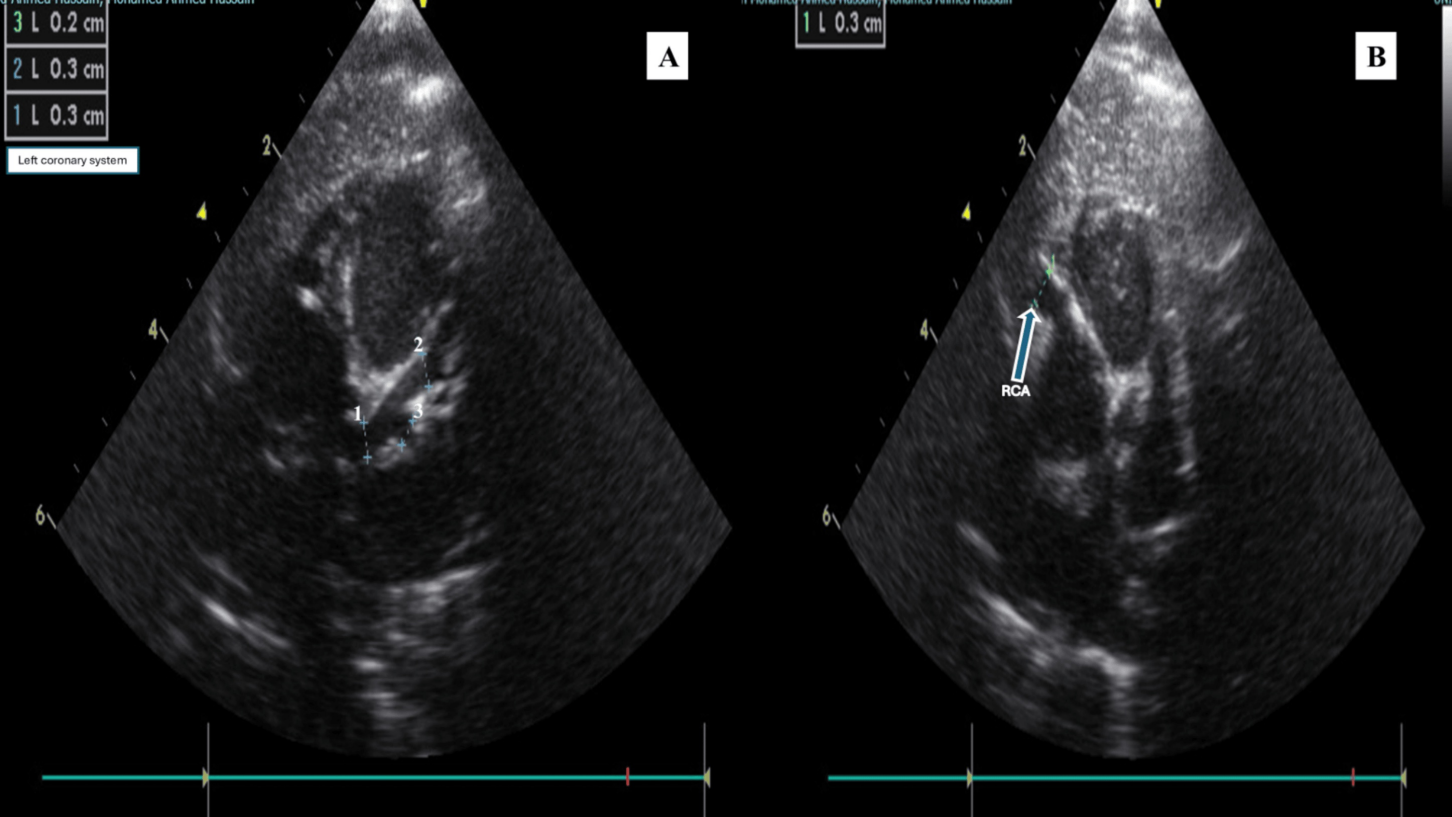
Coronary artery aneurysms at diagnosis on transthoracic echocardiography (A) Left coronary system showing coronary dilation. The on-screen caliper measurements correspond to: 1 = left main coronary artery, 2 = left anterior descending artery, 3 = left circumflex artery. (B) Dilated right coronary artery (arrow); on-screen calipers indicate the measured coronary artery diameter

Procalcitonin remained within normal limits (0.14 ng/mL, reference: <0.5 ng/mL). During hospitalization, a new systolic murmur was noted on cardiovascular examination, prompting cardiac evaluation. Transthoracic echocardiography performed on October 22, 2025 (day nine of illness) demonstrated diffuse coronary artery aneurysms (Figure 1), including dilation of the left main coronary artery (3.0 mm; Z-score +5.5), left anterior descending artery (3.0 mm; Z-score +6.9), left circumflex artery (2.0 mm; Z-score +3.7), and right coronary artery (3.0 mm; Z-score +6.2).

A minimal pericardial effusion was noted, with preserved biventricular systolic function and otherwise normal cardiac anatomy. No structural valvular abnormalities or congenital cardiac defects were identified to explain the systolic murmur. In the context of preserved ventricular systolic function and significant anemia, the murmur was considered consistent with a functional systolic (flow) murmur. Coronary artery dimensions and Z-scores at diagnosis are summarized in Table 3.

**Table 3:** Coronary artery dimensions at diagnosis and follow-up Coronary artery internal diameters (mm) with corresponding body surface area–adjusted Z-scores at diagnosis and follow-up. Coronary artery Z-scores were calculated using the Boston 2017 reference standard, with body surface area calculated using the Haycock formula LMCA: left main coronary artery; LAD: left anterior descending artery; RCA: right coronary artery; LCx: left circumflex artery

Date	LMCA (mm; Z)	LAD (mm; Z)	RCA (mm; Z)	LCx (mm; Z)
22/10/2025 (diagnosis)	3.0; +5.5	3.0; +6.9	3.0; +6.2	2.0; +3.7
07/12/2025 (follow-up; ~6 weeks)	2.5; +3.1	1.3; +0.6	2.0; +2.3	1.5; +1.4

In the setting of persistent fever for approximately eight days, elevated inflammatory markers, marked thrombocytosis, anemia, a negative infectious evaluation, and echocardiographic evidence of coronary artery involvement, a diagnosis of incomplete Kawasaki disease was made. At the time of diagnosis, the patient weighed 4.29 kg. She received a single dose of intravenous immunoglobulin at approximately 2 g/kg (total dose 8 g). Aspirin therapy was initiated at 4 mg orally every six hours (total 16 mg/day). In view of high-risk features, including young age and coronary artery involvement, adjunctive corticosteroid therapy was given as a single 4 mg intravenous dose of methylprednisolone. The patient demonstrated a rapid clinical and biochemical response, with complete resolution of fever and progressive improvement in inflammatory markers.

**Figure 2:**
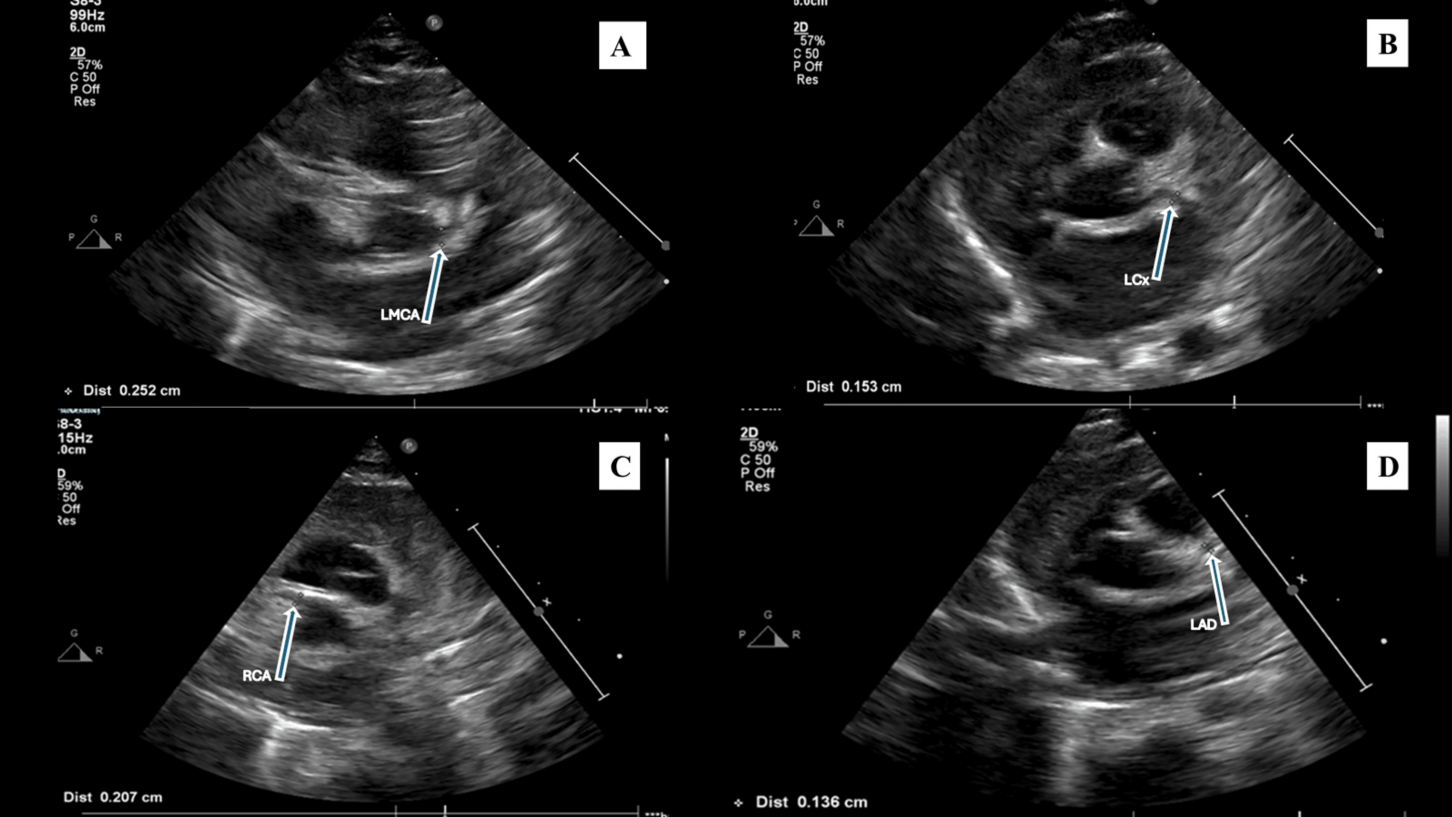
Follow-up transthoracic echocardiography demonstrating coronary artery dimensions after treatment Follow-up images showing the coronary arteries: (A) left main coronary artery, (B) left circumflex artery, (C) right coronary artery, and (D) left anterior descending artery. Arrows indicate the corresponding coronary artery segment, and the on-screen calipers (star-like markers) denote the measured coronary artery diameter

After defervescence, aspirin was continued as low-dose antiplatelet therapy at 12 mg orally once daily during follow-up in the outpatient setting. She was discharged home in stable condition with close cardiology surveillance. Serial outpatient evaluations showed normalization of inflammatory markers, gradual improvement in thrombocytosis and hemoglobin levels, and sustained clinical well-being (Table 1). A follow-up echocardiogram performed approximately six weeks later demonstrated marked improvement in coronary artery dimensions (Figure 2), with a reduction in Z-scores across all affected segments (Table 3). The left main coronary artery remained mildly dilated (2.5 mm; Z-score +3.1), whereas the left anterior descending artery measured 1.3 mm with a Z-score of +0.6, the right coronary artery measured 2.0 mm with a Z-score of +2.3, and the left circumflex artery measured 1.5 mm with a Z-score of +1.4, all reflecting substantial improvement. The patient remained asymptomatic and continues on low-dose aspirin therapy with ongoing cardiology follow-up.

## Discussion

KD is an acute, self-limiting systemic vasculitis that primarily involves medium-sized arteries, with a particular predilection for the coronary arteries, and is therefore a leading cause of acquired heart disease in children [1,2]. Early identification and prompt treatment are crucial, as coronary artery abnormalities, including dilation and aneurysm formation, are key predictors of long-term morbidity and require structured follow-up [1,2]. Diagnostic uncertainty is especially common in infants, among whom KD often manifests as iKD, characterized by fewer than the classic mucocutaneous features, which contributes to delayed diagnosis and an increased risk of coronary involvement [1,2,7,10,11].

This is clinically significant because infants younger than six months consistently demonstrate a disproportionately greater risk of developing coronary artery aneurysms than older children, especially when diagnosis and treatment are postponed [10,11]. Contemporary reviews highlight that the delayed identification of incomplete KD in early infancy continues to pose a significant challenge and is associated with potentially avoidable coronary complications [12]. KD can also present in very young infants, including neonates, in whom incomplete clinical manifestations accompanied by coronary aneurysms have been described, emphasizing the importance of maintaining a high index of suspicion in infants with persistent fever, even at an early age [3].

A key challenge in iKD is its clinical overlap with common infectious syndromes in early infancy. Young infants commonly present with nonspecific upper respiratory symptoms, and clinicians appropriately prioritize ruling out serious bacterial infections, including sepsis, urinary tract infection, and meningitis [7,11]. Consequently, iKD may initially be treated as an infection with empiric antibiotics, thereby delaying consideration of an underlying inflammatory cause [1,3,4,7]. Case-based literature demonstrates that KD in very young infants may occur without the typical constellation of conjunctivitis, oral changes, rash, extremity changes, or lymphadenopathy, and may instead present as persistent fever accompanied by escalating systemic inflammation despite negative culture results and minimal localized findings [3,4]. Reports from the United Arab Emirates (UAE) offer a regional clinical context by describing incomplete KD presentations with coronary involvement [9]. Importantly, similar diagnostic challenges and coronary outcomes have also been documented internationally [3,4,10,11], which underscores the importance of early reassessment and timely echocardiographic evaluation in infants with persistent fever.

Laboratory trends are particularly valuable in suspected iKD because they can offer early supportive clues when the classic clinical features are lacking. The AHA scientific statement outlines a diagnostic algorithm for incomplete KD and emphasizes that increased inflammatory markers, such as CRP and/or ESR, together with supportive laboratory findings, including age-adjusted anemia, leukocytosis, hypoalbuminemia, and thrombocytosis, often emerging after day seven, increase the probability of incomplete KD and inform treatment decisions when other diagnoses have been excluded [1]. The updated 2024 AHA scientific statement reaffirms this framework and includes updated guidance on diagnosis, risk stratification, and management, specifically addressing incomplete presentations in infants [13].

Review literature on iKD similarly highlights that this dynamic hematologic pattern, particularly the development of thrombocytosis in the subacute phase, is typical and may assist in distinguishing incomplete KD from many infectious conditions once cultures remain negative and the clinical response to antibiotics is absent or only partial [7]. Applying the AHA incomplete KD pathway in our patient, the persistence of fever beyond seven days, rising inflammatory markers, anemia, leukocytosis, and pronounced thrombocytosis, together with negative culture results and a reassuring procalcitonin trajectory, closely paralleled the reported laboratory course of incomplete KD and justified broadening the evaluation beyond infection after no alternative source of fever was identified [1,7].

Echocardiography is central to the diagnosis and management of KD, and it becomes even more critical in infants with suspected iKD because coronary abnormalities may be present even in the absence of typical clinical signs [1,6,8]. Contemporary guidance highlights the importance of coronary artery assessment using body surface area-adjusted Z-scores to classify coronary dilation, ectasia, and aneurysms consistently across patient size and age, thereby improving diagnostic precision and informing follow-up planning [6,8]. Z-score-based interpretation is particularly relevant in small infants, in whom absolute vessel diameters can appear modest yet correspond to markedly abnormal Z-scores [6].

In this case, a newly detected systolic murmur prompted echocardiography, which demonstrated coronary artery abnormalities that meet aneurysm criteria by Z-score thresholds, with maximum Z-scores in the medium aneurysm range for left main coronary artery (LMCA) (+5.5), left anterior descending artery (LAD) (+6.9), and right coronary artery (RCA) (+6.2), and small aneurysm range for left circumflex artery (LCx) (+3.7), supporting coronary involvement in incomplete KD [1,6,8]. Because these findings met aneurysm criteria rather than isolated dilation/ectasia, they directly informed risk stratification and supported structured cardiology follow-up with antiplatelet therapy and serial echocardiographic surveillance in accordance with guideline-based management [1,13]. The subsequent reduction in coronary dimensions and Z-scores on follow-up imaging is consistent with expected improvement following timely immunomodulatory therapy in iKD and underscores the value of serial echocardiographic surveillance in this population [1,6,8,10,11].

Differentiating iKD from other inflammatory syndromes is another important diagnostic step. MIS-C may present with fever, elevated inflammatory markers, and cardiac involvement, including coronary changes, and therefore represents an important consideration in the differential diagnosis [5]. However, MIS-C typically demonstrates a broader multisystem phenotype, frequently with prominent gastrointestinal manifestations, myocardial dysfunction, or shock, and it is uncommon in very young infants, which can help distinguish it from KD phenotypes in many cases [5]. The 2024 AHA scientific statement also highlights that myocardial involvement with elevated troponin and/or BNP and depressed left ventricular systolic function is more prominent in MIS-C; therefore, BNP and troponin were obtained in this patient, and while BNP was elevated, normal troponin with preserved systolic function on echocardiography supported MIS-C being less likely in the overall clinical context [13]. Additionally, hematologic patterns can be informative. Thrombocytosis emerging after the first week of fever is more typical of KD, whereas MIS-C more often exhibits thrombocytopenia or differing hematologic trends, reinforcing the importance of integrated clinical, laboratory, and echocardiographic assessment [1,5,7]. In the present case, the clinical phenotype and evolving hematologic pattern, together with echocardiographic coronary involvement, supported iKD as the more consistent diagnosis [1,7].

Intravenous immunoglobulin (IVIG) remains the cornerstone of KD treatment and substantially reduces the risk of coronary complications when administered during the acute phase [1,2]. The AHA scientific statement recommends IVIG at 2 g/kg, ideally within the first 10 days of illness, and supports treatment even beyond day 10 when ongoing inflammation or coronary involvement is present [1,13]. Aspirin is commonly administered alongside IVIG, with many protocols using higher dosing during the acute inflammatory phase followed by low-dose antiplatelet therapy during the subacute period, particularly when coronary involvement is documented [1,2,13]. The primary rationale for continuing aspirin beyond the acute phase is antiplatelet effect and thrombosis risk mitigation in the setting of coronary abnormalities, with dosing and duration tailored according to coronary status and follow-up findings [1,2].

Accordingly, low-dose aspirin was planned to continue until coronary dimensions and Z-scores normalize; given residual LMCA dilation on follow-up, antiplatelet therapy and pediatric cardiology follow-up with serial echocardiography were continued, with interval and duration individualized to residual coronary findings [1,13]. The follow-up goals are to document coronary regression/normalization and tailor antiplatelet therapy duration and surveillance frequency/duration to residual coronary Z-scores, with continued longer-term surveillance if abnormalities persist [1,13]. In our patient, IVIG was administered on day 9 of fever onset, followed by rapid clinical improvement and down-trending inflammatory markers, and follow-up echocardiography demonstrated interval improvement in coronary dimensions, aligning with the expected benefits of timely recognition and guideline-directed treatment in iKD presentations [1-4,10,11].

The role of corticosteroids in KD has evolved, and current guidance supports adjunctive corticosteroids in selected higher-risk patients, including those with early coronary involvement or increased risk of IVIG resistance, as part of intensified initial therapy [1,13]. Reviews of KD management controversies also highlight that practice patterns may differ across institutions regarding the use and intensity of adjunctive therapies, reinforcing the importance of individualized risk assessment and adherence to evidence-informed protocols [12]. In our patient, this risk-based approach was applied, given early coronary involvement in a very young infant, followed by close cardiology monitoring with interval improvement on follow-up echocardiography [13].

This case reinforces that persistent unexplained fever in very young infants, particularly when accompanied by progressive inflammatory markers, anemia, and thrombocytosis, should prompt early consideration of iKD and timely echocardiographic assessment using Z-scores, even when classic clinical criteria are absent, and should lower the threshold for early echocardiography once fever persists beyond approximately one week with elevated inflammatory markers and no alternative diagnosis [1,13]. Early diagnosis and treatment remain essential to reduce preventable coronary morbidity and to guide structured cardiology follow-up based on standardized coronary classification [1,6,8].

Limitations

This report has limitations inherent to single-patient case reports. Firstly, iKD is a clinical diagnosis supported by laboratory and echocardiographic findings, and presentations in early infancy may overlap with infectious syndromes, which can complicate attribution at initial presentation. Second, findings and outcomes from a single case may not be generalizable to broader infant populations or different healthcare settings. Finally, although follow-up echocardiography demonstrated improvement in coronary dimensions, longer-term surveillance is needed to fully characterize coronary outcomes over time, particularly in infants with early coronary involvement.

## Conclusions

iKD in very young infants remains a diagnostic challenge because classic mucocutaneous features are frequently absent, and early presentations can resemble common infectious conditions. This case report of a 2.5-month-old infant illustrates that persistent unexplained fever accompanied by progressive systemic inflammation, anemia, and thrombocytosis should prompt consideration of iKD once alternative etiologies are not supported by microbiologic testing or clinical course. Early transthoracic echocardiography with coronary artery Z-score assessment was pivotal in confirming coronary involvement and establishing the diagnosis despite minimal clinical criteria. Timely administration of intravenous immunoglobulin, with antiplatelet therapy and adjunctive corticosteroid use in a high-risk infant, was followed by rapid clinical improvement and interval regression of coronary dilation on follow-up echocardiography. These findings emphasize the importance of early recognition, guideline-informed evaluation, and structured cardiology follow-up in infants with suspected iKD, particularly in very young infants (≤6 months) who are at higher risk of coronary artery aneurysms, to reduce preventable coronary morbidity and long-term cardiovascular sequelae.

## References

[1] McCrindle BW, Rowley AH, Newburger JW (2017). Diagnosis, treatment, and long-term management of Kawasaki disease: a scientific statement for health professionals from the American Heart Association. Circulation.

[2] Newburger JW, Takahashi M, Burns JC (2016). Kawasaki disease. J Am Coll Cardiol.

[3] Shen M, Liu D, Ye F, Zhang J, Wang J (2024). Kawasaki disease in neonates: a case report and literature review. Pediatr Rheumatol Online J.

[4] Singh S, Inban P, Mishra A (2023). Atypical Kawasaki disease in a 16-month-old baby: a case report and literature review. Cureus.

[5] Karki A, Jha A, Sapkota S, Kashyap N, Manandhar SR (2024). MIS-C-like features in a patient of atypical Kawasaki disease: a case report. JNMA J Nepal Med Assoc.

[6] Kim SH (2022). Diagnosis of coronary artery abnormalities in Kawasaki disease: recent guidelines and Z score systems. Clin Exp Pediatr.

[7] Yu JJ (2012). Diagnosis of incomplete Kawasaki disease. Korean J Pediatr.

[8] McCrindle BW, Cifra B (2018). The role of echocardiography in Kawasaki disease. Int J Rheum Dis.

[9] Al Zubaidi A, Ghatasheh G, Karuppaswamy V, Narchi H (2023). Epidemiology of Kawasaki disease, its incomplete form and outcomes: a single-institution experience in the United Arab Emirates. Cureus.

[10] Salgado AP, Ashouri N, Berry EK, Sun X, Jain S, Burns JC, Tremoulet AH (2017). High risk of coronary artery aneurysms in infants younger than 6 months of age with Kawasaki disease. J Pediatr.

[11] Mastrangelo G, Cimaz R, Calabri GB, Simonini G, Lasagni D, Resti M, Trapani S (2019). Kawasaki disease in infants less than one year of age: an Italian cohort from a single center. BMC Pediatr.

[12] Pilania RK, Bhattarai D, Singh S (2018). Controversies in diagnosis and management of Kawasaki disease. World J Clin Pediatr.

[13] Jone PN, Tremoulet A, Choueiter N (2024). Update on diagnosis and management of Kawasaki disease: a scientific statement from the American Heart Association. Circulation.

